# Electrolithography- A New and Versatile Process for Nano Patterning

**DOI:** 10.1038/srep17753

**Published:** 2015-12-04

**Authors:** Santanu Talukder, Praveen Kumar, Rudra Pratap

**Affiliations:** 1 Centre of Nano-Science and Engineering, Indian Institute of Science, Bangalore 560012; 2 Department of Materials Engineering, Indian Institute of Science, Bangalore 560012

## Abstract

We report a new lithography technique based on electromigration driven material transport for drawing patterns at nanometer scales in ambient conditions. We use a thin metal film as a masking layer and a polymer layer beneath it as a pattern transfer layer. The desired pattern is drawn in the metal layer by etching the metal with a conducting scanning probe assisted by liquid electromigration. The pattern drawn on the metal layer is transferred to the polymer layer by etching the polymer with an appropriate solvent. Subsequently, the pattern is transferred to the desired material layer using a film deposition technique followed by conventional lift-off process. Using this simple technique, we have achieved pattern resolutions of 9 nm on the polymer and 40 nm on transferring the pattern to another material. Based on the ease of use and process costs, this technique promises to be competitive to e-beam lithography that employs high energy and ultra-high vacuum, or the industrial standard ultra-violet light photolithography that employs extremely expensive implements to reach nano-scale resolutions. We also demonstrate direct mask writing using this technique and explain the fundamentals behind the workings of the developed method.

Currently, photolithography is the industry standard patterning technique used for mass scale production of microelectronic devices^1^,^2^,^3^,^4^. Though it is the most reliable and the fastest technique among the existing ones, the achievable resolution is limited, rendering this technique unsuitable for fabricating patterns with nanoscale features^1^,^4^. On the other hand, for nanoscale patterning electron beam lithography (EBL) is the most popular choice^5^,^6^,^7^. However, EBL requires ultra-high vacuum (UHV) and high energy for its basic operation, making the process and the tool very expensive. Moreover, being a direct and serial writing technique, EBL suffers seriously on throughput and, consequently, is far behind the industry standard photolithography. Photolithography, however, requires masks and for preparing masks, we do not have any alternative to direct writing at present. A considerable part of microelectronic production cost goes into writing masks. Hence, a high resolution, low cost, direct writing technique is in high demand in the semiconductor industry. Clearly, any such realization, which is also potentially compatible with the current semiconductor practices, may give a fresh lease of life to Moore’s law.

Naturally, there has been significant effort in inventing alternate lithography techniques or modifying the existing ones^8^,^9^,^10^ and pushing the resolution bar. Nano-imprint lithography (NIL)^11^, dip pen lithography (DPL)^12^,^13^, and scanning probe lithography (SPL)^14^,^15^ came into existence in the recent past as a result of this effort. Nonetheless, as summarized in Table 1, all of these techniques have their own limitations pertaining to either less reliable resolution or limited versatility (i.e., material specific, low thickness patterns, etc.). Thus, an alternate universal lithography technique still does not exist that can be used for both direct writing and preparing masks along with the flexibility of being able to transfer patterns of controllable thickness to desired materials using well established conventional lift-off or etching techniques.

In this work, we introduce a new lithography technique that we call *Electrolithography*. For developing this process, we exploit as well as significantly extend our previously reported^16^,^17^ understanding of controlled nanoscale material transport and pattern formation due to liquid electromigration in thin metal films. We apply this phenomenon to develop a scanning probe based lithography technique where liquid electromigration driven material removal is used as a key step. This is the first time when electromigration, which is essentially a directional material transport phenomenon, and ironically, has often been dreaded as a destructive phenomenon^18^,^19^, has been used for developing a general purpose lithography process. Below, we will firstly describe the fundamentals of electrolithography, including the new and relevant scientific observations related to electric current driven flow in very thin Cr films deposited on an insulating substrate, and then, show and discuss the patterns fabricated using this lithography process.

## Fundamentals of electrolithography

### Liquid Electromigration as Core of Electrolithography Process

Electromigration is an electric current driven, diffusion controlled material transport phenomenon that leads to formation of voids and hillocks in a solid metallic conductor near cathode and anode, respectively^18^,^19^. Recently, some studies on the electromigration driven flow of liquid metals over a relatively long range have also been reported^16^,^20^,^21^,^22^. Similar to the electromigration in solids, electromigration driven mass transport in liquid metals also varies linearly and exponentially (Arrhenius type) with the applied current density (or, electric field) and the temperature, respectively^20^,^22^. However, unlike the solid state electromigration, the direction of the material transport in the liquid materials is not always from the cathode to the anode: some liquids, such as liquid Ga, Sn, Al, etc., flow from the anode to the cathode (i.e., in the direction of electric field) whereas a few other liquids, such as liquid Pb, flow from the cathode to the anode (i.e., in the direction of electron flow)^22^.

**Figure 1:**
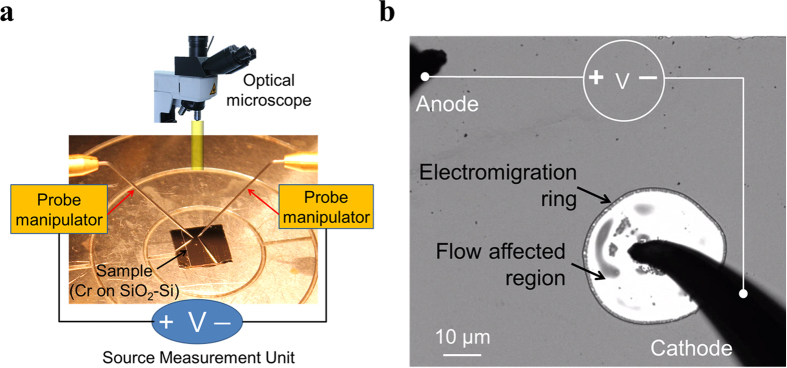
Electromigration induced radially symmetric flow. (**a**) Schematic illustration of the experimental setup for passing electric current through a thin Cr film deposited on a substrate. (**b**) An optical image showing formation of a typical electromigration ring around the cathode probe (solid black parts are needle probes). The ring is created on a 20 nm Cr film deposited on SiO_2_-Si substrate (Refer to Supporting Information for the video attached to this figure showing real time flow process).

In the particular case of an infinite Cr thin film deposited on an insulating substrate, the application of a high electric current using two probes situated sufficiently far away from each other (see Fig. 1a for experimental set-up) and, in the presence of air, results in liquefaction of material below the cathode, which then starts to flow in a radially symmetric fashion away from the cathode probe (Refer to Supporting Information for a video showing this phenomena)^16^. Figure 1b shows a snap-shot of the electric field induced liquid material flow in a 20 nm thick Cr film deposited on SiO_2_-Si substrate upon passing a current of density ~10^9^ A/m^2^ through a probe tip. This corresponds to a current density of ~3.3 × 10^9^ A/m^2^ in the thin Cr film at a distance of 10 μm away from the probe tip. Such a flow is attributed to electromigration because (i) the observed material flow is directional (i.e., it occurs at only one of the two electrodes), (ii) directionality of the flow depends on the material (e.g., the flow occurs from the cathode probe for Cr and from the anode probe for Al), (iii) flow occurs along or in the reverse direction of the electric field lines, and (iv) the dependence of flow rate on the electric current and the temperature are linear and exponential, respectively, as predicted by the standard electromigration theory^16^,^17^.

**Figure 2:**
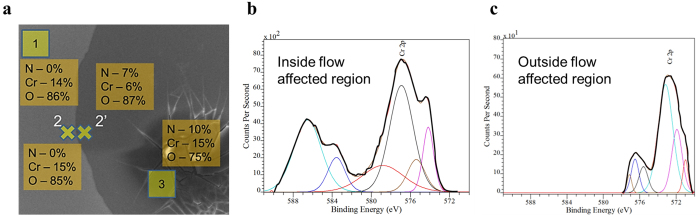
Elemental analysis of the regions inside and outside of the flow affected region. (**a**) Atomic percentage of nitrogen, chromium and oxygen in different regions. Area 1 and area 3 are completely outside and inside the flow affected region, respectively, whereas spots 2 and 2’ are marginally outside and inside of the periphery of the flow affected region, respectively. Area-scans were conducted in the rectangular zones marked as 1 and 3 whereas point-scans were conducted at the centers of the cross-marks denoted as points 2 and 2’. High-resolution XPS spectra of Cr 2P orbital (**b**) inside and (**c**) outside the flow affected region. Thick black line is the actual data and the thin lines are the fitted curves.

Chemical analysis of the flow affected region (see Fig. 1b) was performed using energy dispersive spectroscopy (EDS) and X-ray photo-spectroscopy (XPS). Figure 2a shows the composition in atomic percentage of chromium, oxygen and nitrogen inside and outside the flow affected region as evaluated using EDS. Chromium and oxygen are present at both the inside and the outside the flow affected region and their relative concentration do not vary due to the formation of electromigration ring or flow affected region. However, interestingly, nitrogen is present inside the flow affected region only. So, it can be inferred that chromium compound having nitrogen in it is present only inside the flow affected region.

Figure 2b shows the XPS spectra of the flow affected region. Cr(NO_3_)_3_ peak is observed at 577.3 eV inside the flow affected region. For comparison, XPS analysis was also conducted outside the flow affected region, as shown in Fig. 2c, for the same sample. No peak is observed at and above 577 eV in this case. So, it is concluded that Cr(NO_3_)_3_ is formed only in the flow affected region and there is no nitrate compound in the pristine Cr film. Thus, Fig. 2 clearly shows that the flow affected region constituted of Cr(NO_3_)_3_ compound. All the peaks in Fig. 2c, i.e., outside the flow affected region, as well as all peaks in Fig. 2b except 577.3 eV peak, i.e., inside the flow affected region, represent various chromium oxides.

Furthermore, the sample with the flow affected region was heated in air by placing it on a hot plate kept at a temperature of 60 to 65 °C. Such heating results in melting in the flow affected region, again, confirming the presence of a Cr compound with a melting temperature of only 60 °C, which is the known melting temperature of hydrous Cr(NO_3_)_3_. Thus, we conclusively show that the liquid material flowing away from the cathode is the low melting temperature Cr compound, namely Cr(NO_3_)_3_. The current densities passing through the cathode probe are sufficient to melt Cr(NO_3_)_3_ whose melting temperature is only 60 °C. It should be categorically noted that such a flow neither ensued in the absence of an electric field nor in the absence of air (mainly, N_2_ and O_2_) or in the vicinity of the anode probe. Thus, nitridation precedes the electric current induced flow.

### The Electrolithography Process

**Figure 3:**
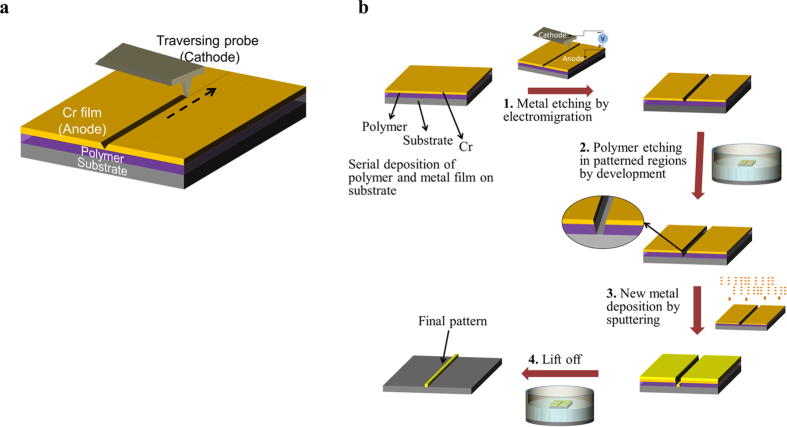
Important steps of the standard electrolithography process. (**a**) Electromigration driven metal etching by a traversing probe: As the negatively biased tip moves, the Cr compound formed below the cathode melts and flows away from the path creating a groove along the path traversed. The dashed arrow shows the direction in which the tip is traversed. (**b**) Process flow of the standard electrolithography technique: The process starts with a substrate spin-coated with a polymer followed by the deposition of a top layer of Cr thin film. (1) In the first step, the top Cr layer is etched in the desired pattern using electromigration. (2) Next, the polymer is etched in the patterned region by dipping it in an appropriate solvent. The inset shows the zoomed view of the trench made in the polymer. (3) Subsequently, the desired material is deposited, and (4) lift-off is used to transfer the final pattern on the desired material.

Figure 3 schematically shows the electrolithography process that we explain here step by step. As mentioned earlier, in an infinitely extended thin film of Cr, the molten Cr compound flows radially outward from the cathode probe creating a ring shaped pattern around the static probe (see Fig. 1b). However, if the probe is moved on a path, linear or curved, while keeping the sample at electrical ground and the tip at a negative bias, the radial symmetry of the flow breaks and the liquid material flows away from the path on either side of the traversing probe creating a trench pattern along the way. Figure 3a,b(1) schematically show this metal etching process.

After etching away the metal by liquid electromigration, the polymer layer deposited in between the substrate and the metal layer gets exposed in the etched region and the exposed polymer is subsequently etched away using an appropriate solvent (Fig. 3b(2)). The remaining top layer of Cr inhibits the removal of polymer layer beneath it. Following the removal of the polymer from the etched or trenched region exposing the substrate, a new material can be deposited on the substrate in the exposed region using standard thin film deposition techniques (Fig. 3b(3)). Once the film is deposited or pattern is transferred to the desired material, a standard lift-off process using another solvent (stronger than previously used) is used to remove the remaining polymer along with the top layer of the thin Cr film (Fig. 3b(4)).

## Results

### Standard Electrolithography

**Figure 4:**
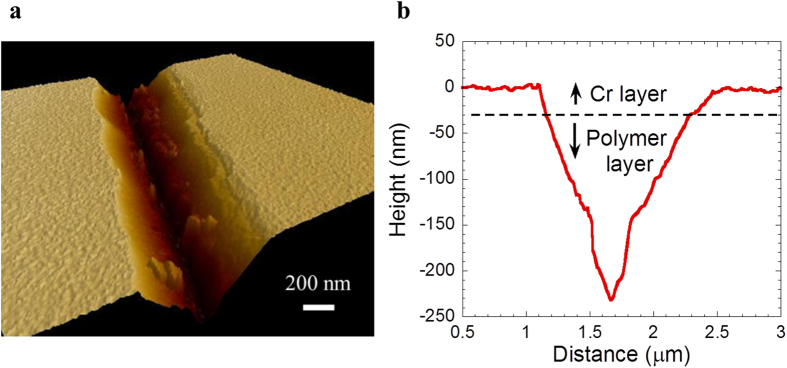
Transferred etch pattern in the polymer layer below the metal film. (**a**) 3-D AFM image of the patterned region after polymer etching, and (**b**) Cross-sectional profile of the etched groove in the polymer.

Figure 4 shows the etch profile created in polymethyl methacrylate (PMMA) (the polymer used here) following the etching of PMMA by acetone below the etched Cr film. As shown in Fig. 4, the polymer etch profile is generally ‘V’ shaped, albeit with a flat bottom. This is attributed to the isotropic etching of PMMA which ensures more lateral etch in the top region than at the bottom because of the greater exposure time of the top in the solvent. However, as will be discussed later in detail, the “V” shape of the etch profile offers an additional control, besides the tip diameter of the probe, the electric current, the tip velocity, developing time, and the lift-off process, for controlling the width of the finally deposited material. Figure 4b, which shows the formation of a 230 nm deep trench in 30 nm thick Cr and 200 nm thick PMMA, confirms that the entire PMMA in between the Cr top layer and the substrate has been removed following the polymer etch step. Interestingly, no such trenched pattern is created if the atomic force microscope (AFM) tip is traversed with the same contact force but without applying an electric field between the tip and the Cr film, thus, confirming the vital role of electromigration induced material transport in this process.

**Figure 5:**
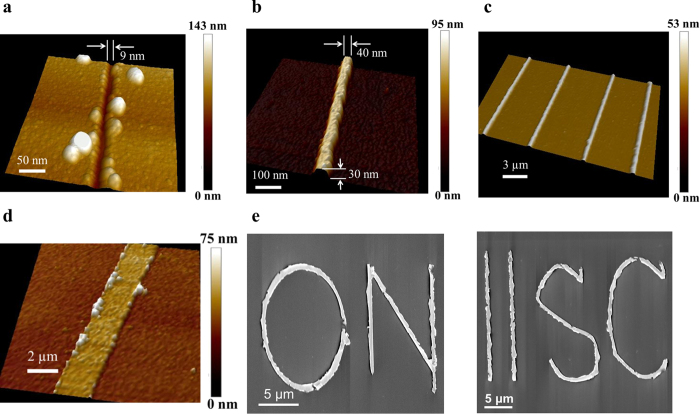
Various patterns created using electrolithography. (**a**) The narrowest trench in PMMA with a channel width of 9 nm. (**b**) The narrowest Ti thin film line with an average width of 40 nm and thickness 30 nm. (**c**) A set of 100 nm wide and 40 nm thick parallel Ti lines deposited on Si substrate. **(d)** A 3 μm wide Ti line fabricated on Si substrate. Instead of using an AFM setup (as used for figures (**a,c**)), this pattern was created using a probe-station setup with manual XYZ movement control. A tip of 2 μm diameter was used for writing this pattern. (**e**) Images of various types of patterns fabricated in Au on Si substrate. Figures (**a–d**) are AFM images whereas the set of images shown in (**e**) are scanning electron microscope (SEM) micrographs. Any pattern similar to (**a**) was not formed if no electric field was applied between the tip and the top metal layer.

Figure 5a shows an AFM scan generated 3-D view of the narrowest trench obtained in the polymer layer. A similar opening in the polymer layer was used to deposit the narrowest Ti thin film shown in Fig. 5b. Following an optimization exercise involving various aforementioned electrolithography controls, we were able to achieve absolute widths of 9 nm in the PMMA and 40 nm at the base of the transferred metallic pattern (see Fig. 5a,b, respectively). These remarkable lateral resolutions were achieved while the entire etching process was conducted under the ambient conditions and the final Ti film was deposited using the standard RF sputtering.

Figure 5c shows an example of multiple parallel straight lines of ~30 nm thick Ti lines on Si substrate created simultaneously using the electrolithography process. The absolute width at the base of each Ti line is ~100 nm. The electromigration induced etching of the straight-line patterns in the Cr top layer was conducted sequentially using an AFM tip whereas the rest of electrolithography process (i.e., steps shown in Fig. 3c(2)–c(4)) for all lines were performed simultaneously. Creation of identical parallel straight lines confirms the stability of the etched patterns during subsequent processing as well as repeatability of the electrolithography process.

Figure 5d shows micrograph of a very wide (3 μm) straight line of Ti thin film. Figure 5e shows a few different patterns involving straight and curved lines, open and closed shapes, as well as shapes with round and sharp edges of Au thin film, fabricated on Si substrate. The electromigration induced etching of Cr film for fabricating the pattern shown in Fig. 5d was carried out using a probe of 2 μm diameter while the patterns shown in Fig. 5e were created using an AFM tip. Figure 5 readily reveals the following salient features of electrolithography: (i) it is a highly scalable process which can be used to prepare patterns with width ranging from a few tens of nanometers to a few micrometers, and (ii) it can be used to fabricate simple as well as complex shapes.

Patterns shown in Fig. 5c,e are 40 and 75 nm thick, respectively, whereas the highest resolution line, as shown in Fig. 5b, is 30 nm thick. Thus, a high etch depth in the polymer layer ensures that the patterns drawn are thick enough to be used for practical applications in electronic circuits.

The patterning described above is performed using an additive process (i.e., depositing a material in the desired pattern over the substrate). We can also use electrolithography for subtractive patterning by removing material from the metal layer for direct patterning on a substrate and one-step mask writing on transparent substrate, as described below.

### Materials Removal Mode of Electrolithography

**Figure 6:**
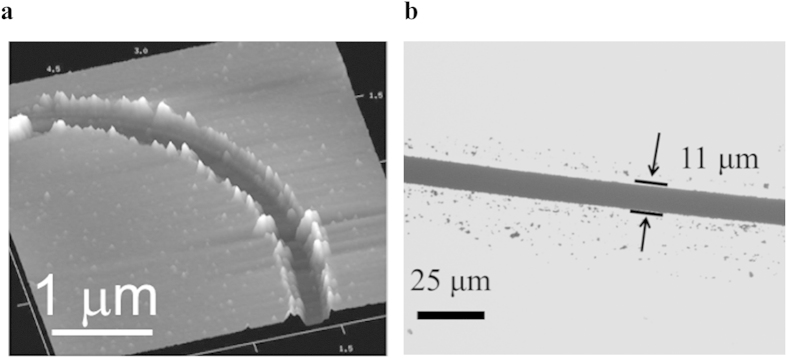
Material removal mode of electrolithography. (**a**) 3-D AFM micrograph of an arc written on a glass substrate by etching away Cr from the exposed region. A blanket layer of 50 nm thick Cr film is deposited on the glass substrate which is subsequently removed leaving a negative of the pattern formed by electromigration induced etching. Also, a blunt AFM tip of radius 1.6 μm is used for electromigration induced etching which finally produces a line-width of 500 nm on the glass substrate. The white regions at the edges of the arc are leftover PMMA which were not completely removed during the second stage of developing. Although this residual layer was successfully removed from several locations using RIE, either longer periods of RIE or some other combination of polymer and reagent during lift off should be explored to attain a residue free pattern. (**b**) Direct patterning on transparent glass by electromigration induced etching of Cr film: In the shown optical image, the darker region is transparent as the Cr has been etched away from those regions.

To demonstrate the material removal mode of electrolithography, we need to modify the sample configuration shown in Fig. 3 slightly by placing an additional layer of the desired material (Cr thin film in this case) in between the PMMA and the substrate. Once the PMMA below the electro-etched top Cr layer is etched away during the first stage of polymer etching, the desired material to be patterned just beneath the trench becomes exposed (see Fig. 3b). Subsequently, this exposed layer of material is etched using a suitable etchant (e.g., a HClO_4_ and (NH_4_)_2_[Ce(NO_3_)_6_] based etchant for Cr used here). Since the material in the non-patterned region is still protected by the polymer (PMMA in this case), the material layer in the exposed regions only gets etched by the etchant. Following the etching of the material, the newly exposed or created space can even be filled with another material if desired, forming a multi-layered composite thin film structure on the substrate. Once the desired pattern is formed, the remaining PMMA and the top Cr layer can be removed using the standard lift-off process. The arc shown in Fig. 6a is etched in the final Cr layer deposited on the glass substrate using the described process. The ability of electrolithography to operate in both additive and subtractive modes proffers possibility of using it in multi-level, multi-step processes, as often required in semiconductor practices.

Besides the method of material removal from the substrate as explained above, the direct electromigration induced etching of opaque films of Cr deposited on a transparent glass substrate can also produce masks for photolithography. Thus, such mask writing becomes a one-step process with electrolithography. To explore this aspect of the electromigration induced etching and electrolithography, we deposit a 50 nm thick Cr film on a glass substrate without using any polymer layer in between. Subsequently, the Cr film is etched selectively using electromigration leaving these regions optically transparent. Figure 6b shows one such instance. Although we have successfully fabricated patterns using these electro-etched masks, we could achieve only the best lateral resolution of a few micrometers as of now. The poor resolution for direct mask-writing on glass, as compared to the example shown in Fig. 5, is attributed to the excellent wetting between the liquid Cr compound and the glass substrate.

## Discussion

### Parameters Affecting Resolution

Resolution — the minimum width of the patterns — is determined by the geometry of the ‘V’ shaped profile etched into the polymer layer. As shown in Supporting Information, the resolution, *W*_B_, and the inclination of the ‘V’ profile, 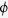, can be given as:


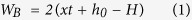



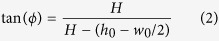


where *x* and *t* are etching rate of polymer and time for etching, respectively, *h*_0_ and *H* are the initial penetration in polymer and the total thickness of the polymer, respectively, and *w*_0_ is the etch width on the top metal layer (see Fig. S1 in Supporting Materials).

Now, *h*_0_ and *w*_0_ depend on the tip diameter and the electromigration process parameters, such as current (or voltage), force applied on the tip, etc. In general, increasing the tip diameter and the force applied to maintain the contact between the tip and the substrate (deflection voltage on the AFM tip) increases both *w*_0_ and *h*_0_. Also, *w*_0_ increases with the current or voltage applied during electromigration. Such understanding of the process parameters on resolution allows us to prescribe optimization of the resolution for a given pattern. For example, *W*_B_ increases linearly with *h*_0_ whereas the denominator of equation (2) becomes smaller and hence 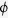 gets closer to 90^0^ with an increase in *h*_0_. Thus, better resolution and steeper sidewalls for the final patterns have contradictory requirements on *h*_0_ or the force applied during electromigration necessitating determination of an optimum by plotting equations (1) and (2) as functions of *h*_0_. Nevertheless, various parameters in equations (1) and (2) cannot be arbitrarily affixed due to several physical limitations. For example, none of the currently available tips can remain atomically sharp over continued “in-contact” usage (see next section for more discussion on it). Hence, there is a need to prepare conducting tips of extremely hard materials which can overcome not only the above limitation but also decrease the lower limit of patterns formed using electrolithography. Furthermore, the finite thermal conductivity of the top metal layer and the kinetics of associated phase transformations place a constraint on the maximum tip velocity and hence the overall throughput.

### Comparison with Other Lithography Processes

**Figure 7:**
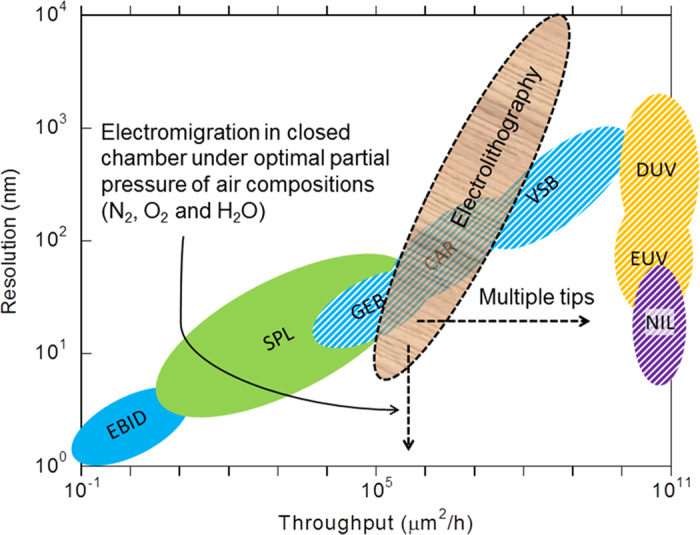
Resolution versus throughput map showing the relative location of various lithography processes. The basic framework of this plot is taken from *(15)*. The slanted hatching indicates a versatile process. The dashed arrows show the steps required to move the envelope of electrolithography towards the right-bottom corner. As shown in Supporting Information, the throughput for electrolithography is of the order of 10^5^–10^9^ μm^2^/h.

Figure 7 (redrawn from ref. 15) shows the relative location of our process with respect to different existing lithography processes, on a map of resolution versus throughput. Figure 4 also uses slanted hatching for identifying versatile processes that can be used to deposit films of high thickness and are not confined to patterning specific materials.

As shown in Fig. 7, there are currently a few lithography processes that give better resolution or better throughput compared to electrolithography, and, electrolithography is collocated with Gaussian electron beam (GEB) and chemically amplified resist (CAR) techniques. However, for the same resolution, electrolithography can provide at least one order of magnitude higher throughput than GEB and for the same throughput, it can provide significantly better resolution as compared to CAR.

EBID and SPL are the two main techniques that give better or equivalent resolution compared to electrolithography. EBID is a material additive process. A precursor compound containing the material to be deposited is inserted in the e-beam chamber in the gaseous state. The compound decomposes in presence of a focused, high energy e-beam and forms a non-volatile product on the substrate as per the desired pattern. Figure 7 indicates that contrary to electrolithography, EBID and SPL are not versatile as they are either material specific or suffer from extremely limited thickness of the transferred patterns (also, see Table 1). In case of EBID, it is also difficult sometimes to find a suitable precursor, which can be easily transferred to gaseous state by vaporization or sublimation. Furthermore, the throughput of electrolithography is orders of magnitude higher than these two techniques. It should be noted that optimization of the electrolithography process may significantly improve its resolution, making this technique a potential candidate for fabricating patterns which are currently created using EBID and SPL.

We again refer to Fig. 7 to show that while Variable Shaped Beam (VSB), Deep Ultraviolet (DUV), Extreme Ultraviolet (EUV) and NIL can provide higher throughput than electrolithography, the achievable resolution using any of these techniques is generally inferior to that of electrolithography. Similar to EBID, VSB also requires high voltage electron beam source and UHV condition, which are not required for electrolithography. DUV and EUV lithography can produce high resolution features with the help of CAR technique. However, electrolithography can produce the same or better resolution by using any polymer and without any UV source. Even green polymers and developers, such as silk and water, respectively, can be used in electrolithography process^23^. Although NIL gives better resolution and throughput, it requires a mask or mold for writing. Thus, unlike electrolithography, NIL cannot be used for direct writing.

Electrolithography is a unique process, where patterns are directly written on a metal film in *material removal mode*. Other well-known processes used for writing directly on a metal film mostly act in the *material addition mode*, e.g., anodic oxidation and dip pen lithography. However, pattern transfer is usually difficult in material addition mode. In addition, the SPL techniques which work in material removal mode, such as thermal SPL or chemical SPL, can write only on polymers. Electrolithography can be used to directly write patterns on Cr without using any resist or polymer. Interestingly, Cr thin film is used as the masking layer in photolithography masks. Hence, electrolithography has potential to be used for resistless direct mask writing also, making it attractive for practices relying on photolithography, such as semiconductor processing.

Electrolithography is one of the few lithography processes that are carried out at room temperature and atmospheric pressure. Such features of electrolithography in addition to usage of green polymers, non-toxic solvents, low energy and small footprint make it a very low cost and environment friendly process. Furthermore, electrolithography can be used to transfer high depth profiled patterns to any material. The scalability associated with electrolithography is very high; that is, the same process as well as the setup can be employed for fabricating patterns of nanometer to micrometer sizes.

### Roadmap for Future Development

Most of the process steps of electrolithography are performed in conventional EBL and photolithography also. Only difference is that while e-beam and UV light are used in EBL and photolithography, respectively, for writing patterns, electrolithography uses an electrically biased scanning tip to create etch patterns. Thus, in principle, there should not be any major concern in using electrolithography for fabricating devices that are currently fabricated by photolithography or EBL. Electrolithography, in both additive and subtractive modes, can potentially be also used for multi-level patterning if robust and accurate control for tip movement, current flow, and other associated processes can be achieved. However, electrolithography involves deposition of a metal layer on top of a substrate or a polymer layer, making this process slower than processes not involving this extra step, such as UV photolithography, etc. In addition, electrolithography relies on scanning probe based process and hence it is inherently slower as compared to photolithography. However, speed of this technique can be improved by parallel writing using multiple probes. Therefore, before electrolithography can be completely integrated into semiconductor practices, compatibility checks and further development are warranted.

Despite of its various advantages, electrolithography, in its current development state, cannot produce arbitrarily small pitch. As the tip traverses, material is removed forming a trench in Cr film and the removed material piles-up at the “bank” of the trench. Thus, the minimum pitch that can be attained in the Cr film is equal to the width of an etched trench. Besides this physical limitation, since we are dealing with metals and are at liberty to choose electric current insensitive polymers, dynamic optimization of electric current values may limit other proximity effects, such as wetting, edge effects, etc. In addition, as mentioned earlier, this process currently uses a wet-etching step (developing stage), which is isotropic in nature, for removing polymer from the patterned regions. Thus, the polymer is etched in all directions equally, leading to broadening of the top of the polymer layer. This broad opening at the top of the PMMA layer further limits the pitch. However, this limitation can be overcome by using anisotropic dry etching techniques, such as reactive ion etching (RIE), in place of the isotropic wet chemical etching.

Electrolithography is a contact mode process. Hence, the geometry of the writing tips degrades with time. The rate of tip degradation depends on various process parameters, such as current, deflection voltage, scan speed, ambient conditions, tip-metal interaction, etc. At the beginning, the diameter of a new tip is usually 10 nm, which, after first few writings (approximately 1000 μm long vector scan), increases to 100 nm. However, after the initial degradation the diameter of the tip remains 100 nm for a long time and successfully produces identical patterns (as shown in Fig. 5c). It should be noted that the tip diameter alone does not affect the patterns resolution. The resolution depends on the opening at the bottom of the polymer layer, which along with the tip diameter, depends also on the polymer etching step, the electric current, and the velocity of tip. Nevertheless, degradation of the tip is a consumable cost associated with this technique and some innovative solutions, including control of environment, tip with composite or graded microstructure, top metal (to be etched) specific tip material, etc., for reducing the tip degradation must be found before this technique can be commercially competitive.

Thus, electrolithography is a novel versatile lithography process which is still in incipient stage. As mentioned above, it has a lot of room for improvement and fine tuning. As shown in Fig. 7, it can easily be modified by incorporation of multiple, independently moving tips for high throughput and optimization of its environment for more finely controlled electromigration leading to better resolution. The chemistry of the polymer and the solvents can be further improved for edge retention, shorter processing time, and environment friendliness. Since electrolithography can be conducted in ambient conditions where handling of liquid chemicals used for etching at different stages is easy, its miniaturization and integration with a thin film deposition system can lead to realization of a small footprint, table-top lithography-cum-film deposition integrated tool or a modular fabrication system. Also, since the electrolithography technique is a scanning probe based technique, it may be seamlessly integrated with other SPL techniques, such as thermal scanning probe lithography, etc., to produce a hybrid lithography technique with minimal limitations.

In summary, electrolithography is an electromigration driven scanning probe based highly scalable low cost lithography process which can be used in ambient conditions. It has the potential of becoming a universal lithography technique, with optimum and wide range of combinations of resolution, throughput and versatility that can achieve the “sweet spot” of the right-bottom corner in Fig. 7 through further developments as outlined above.

## Materials and Methods

### Polymer spin coating

First, a polymer layer of polymethyl methacrylate (PMMA) is spin coated on a clean Si or glass substrate. PMMA is chosen here as it is one of the most widely used polymers in various lithography processes. Different concentrations of PMMA and rotational speeds are used to achieve different thicknesses of the coated polymer layer. For example, 2 and 4% of solid PMMA in anisole solvent spin coated at 6000 rpm give polymer thicknesses of about 65 and 200 nm, respectively. Two different molecular weights (495 and 950 g/mol) of PMMA are used in this process to evaluate the effect of molecular weight and length of the polymeric chain on the edge retention during various developing stages. After spin coating, the sample is heated at 180° C for 2 minutes for hardening the polymer layer.

### Cr thin film deposition

Subsequently, a Cr film of thickness 10 nm to 30 nm is deposited over the PMMA polymer layer by radio frequency (RF) magnetron sputtering. The range of 10 to 30 nm thickness for the Cr film is chosen because: the liquid Cr compound flow velocity is low for low thickness films^17^, which makes the electromigration induced etching process more controllable. However, very thin films are highly resistive wherein, while operating under constant voltage mode, the current becomes too low to melt the material and ensue the subsequent flow. From experimentation, 10 to 30 nm film thickness is found to be the optimum range for this process. We have used 20 nm thick films in most of the samples reported here.

### Electromigration induced writing

For passing the required current as well as drawing patterns, an atomic force microscope (AFM) unit is used in anodic oxidation mode. In this mode, xyz movement of the tip is precisely controlled. The tip voltage can be varied from –10 to +10 V, and the sample is always maintained at the ground potential. For electrolithography, a negative bias of 2 to 4 V is applied on the tip. Material removal is not effected properly below –2 V whereas it is very difficult to control the liquid material flow at high voltages (>5 V).

It is important to note that since the metal layer acts as the terminal in the path of electron flow towards the substrate, the polymer beneath this thin metal layer is not exposed to any significant electric field. Hence, the chemistry of the polymer layer does not get affected during electromigration induced etching, except for the effect of Joule heating induced additional hardening of the polymer. Since the increase in the temperature of the polymer layer during electromigration assisted etching process may cause local expansion in the polymer layer in the vicinity of the etch pattern, the resulting thermal strain may result in distortion of the overall structure. However, given the small volume of expanding polymer (as the thermal conductivity of polymers is often very low) and its low stiffness, issues pertaining to thermal strain mismatch were not critical in patterns created in this study. Nevertheless, creating patterns of ultra-high resolution, especially on soft substrates, which may require very thin metal layer, may be a challenge and will require further optimization.

A vector scanning process, where the tip is moved with a constant velocity to write the pattern, is followed in this work. Hence, the writing area is linearly proportional to the writing or etching time.

For performing electromigration induced etching, the force exerted on the AFM cantilever should be enough to ensure contact between the tip and the sample. In addition to the required force to maintain this contact, the applied force is increased by 5 to 10% in order to push the probe a little into the polymer layer, thereby, confirming that the material removal is proper and no droplets or residues of the liquid material are left on the path. The deflection voltage and the cantilever stiffness determine the contact force. During the electrolithography process, a deflection voltage ranging from 1 to 5 V is applied, which corresponds to 10 to 40 μN contact force, depending on the cantilever stiffness and the deflection sensitivity. However, it should be noted that while applying the same deflection force without any tip voltage, no trench pattern is formed on the Cr film.

### Wet etching of polymer

Once the metal is etched away, the polymer is exposed in the patterned region while it is protected by the metal film in the un-patterned regions as shown in Fig. 3b(1). Dipping the sample into a suitable solvent etches the polymer, thus transferring the patterns to the polymer layer. Methyl isobutyl ketone (MIBK), isopropyl alcohol (IPA) or acetone can be used to etch the PMMA layer. Depending on the molecular weight of the PMMA and the pre-baking temperature, different solvents give different etching rates. For example, 100–120 seconds are required for acetone to etch away 220 nm thick PMMA 950 A4 layer whereas approximately 120–180 seconds are required for MIBK to etch 65 nm thick PMMA 495 A2 layer. Here, A2 and A4 represent 2 and 4% of solid PMMA, respectively, dissolved in anisole solvent.

### Pattern transfer by lift off

After polymer etching, patterns can be transferred to other materials by adding or removing materials in the pattered region. Here, we demonstrate both these techniques successfully. For additive technique, we deposit different metals such as Ti and Au by RF magnetron sputtering, and then develop the patterns using conventional lift off. For lift off, the sample is dipped in acetone and sonicated for 3–4 minutes to remove the PMMA and the Cr layer over it from the un-patterned regions. For a few samples, reactive ion etching (RIE) is done for two minutes in presence of oxygen plasma to remove the residual PMMA sticking at the edges of the deposited patterns.

## Additional Information

**How to cite this article**: Talukder, S. *et al.* Electrolithography- A New and Versatile Process for Nano Patterning. *Sci. Rep.* **5**, 17753; doi: 10.1038/srep17753 (2015).

## Supplementary Material

Supplementary Video 01

Supplementary Material

## Figures and Tables

**Table 1:** A comparative assessment of various existing lithography techniques.

Technique	Major Capabilities	Major Limitations
NIL^11^	^•^ Very high throughput* ^•^ Good resolution (~25 nm)	^•^ Reliability issues arising due to sticking and thermal expansion of polymer imprint resist ^•^ Indirect writing process; requires a mask
DPL^12^,^13^	^•^ Direct writing process ^•^ High resolution ^•^ Suitable for some specific applications, e.g., functionalization of a surface for biological and chemical sensing	^•^ Only a positive writing process# ^•^ Low throughput ^•^ Material specific ^•^ Usually limited to a pattern thickness ≤ 50 nm ^•^ Requires humidity and temperature controlled chamber for high resolution
SPL^14^,^15^,^24^,^25^,^26^,^27^,^28^,^29^,^30^	^•^ Direct writing processes ^•^ High resolution ^•^ A gamut of thermal, mechanical, chemical and electrical effect based processes, e.g., anodic oxidation, dynamic ploughing, bias assisted patterning, etc., proffering customization	^•^ Low throughput ^•^ Material specific$ ^•^ Difficult to transfer patterns to other materials by conventional lift-off or established etching techniques ^•^ Final patterns are extremely thin ( ≤ 20 nm); not suitable for conducting electric current§

^*^~10^12^ μm^2^/hour, equivalent to writing 30 numbers of 200 mm wafers per hour.

^#^Mass addition process where the writing material is directly deposited from the tip to the substrate for pattern printing.

^$^For example, anodic oxidation can be used to pattern SiO_2_, but cannot be used to pattern Si_3_N_4_ or Au lines.

^§^Lines with thickness > 50 nm are preferred in electrical circuits.
