# Supplementary material for: Electrolithography- A New and Versatile Process for Nano Patterning
Source: Sci Rep. 2015 Dec 4;5:17753. doi: 10.1038/srep17753 (PMC4669457; doi:10.1038/srep17753)
Supplement: Supplementary Material [file srep17753-s2.doc]

**Electrolithography- A New and Versatile Process for Nano Patterning**

Santanu Talukder^1^, Praveen Kumar^2,*^ and Rudra Pratap^1^

^1^Centre of Nano-Science and Engineering, Indian Institute of Science, Bangalore 560012.

^2^Department of Materials Engineering, Indian Institute of Science, Bangalore 560012.

Correspondence and requests for materials should be addressed to P.K. (E-mail: [praveenk@materials.iisc.ernet.in](mailto:praveenk@materials.iisc.ernet.in)).

**Supplementary Material**

**Supplementary material 1. Resolution of the scribed pattern**

Resolution — the minimum width of a patterned line — and pitch — the spacing between two adjacent patterns — are determined by the geometry of the ‘V’ shaped profile etched into the polymer layer. We now examine the geometry of the profile and its controllability with the help of Figure S5 that shows a schematic of a typical etch profile.


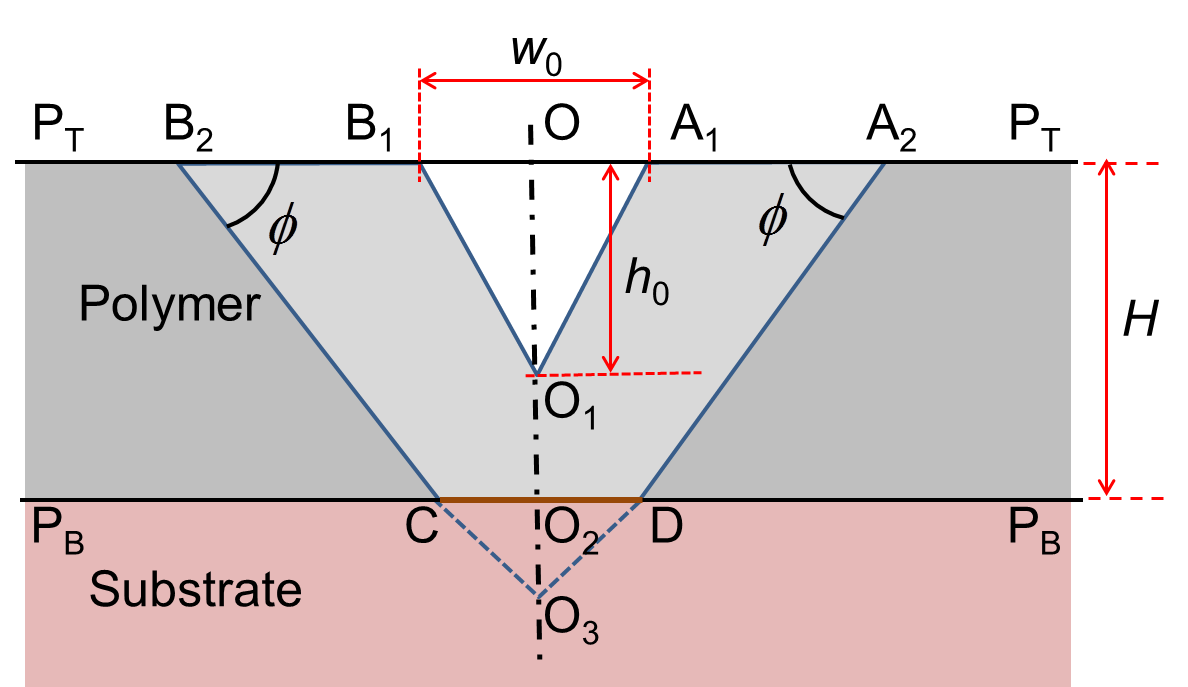


**Figure S1:** **Schematic of the cross-section of an etch profile before and after the first stage of etching of the polymer layer**. P_T_P_T_ and P_B_P_B_ represent the top and the bottom layers of the polymer, respectively, whereas the triangle A_1_B_1_O_1_ and trapezium A_2_B_2_CD represent the initial and the final etch profiles, respectively.

In Fig. S1, the triangle A_1_B_1_O_1_ represents the profile of the groove created in the PMMA during electromigration induced etching. The initial opening in the metal layer, *w*_0_, depends on the tip voltage and the tip diameter. Now, since this etching process is isotropic, the opening at the top of the polymer as well as the etch depth increase with time, forming first a ‘V’ shaped and then a ‘
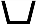
’ shaped (open trapezium) (A_2_ B_2_CD in Fig. S5) etch profile in the polymer. The ‘V’ shaped profile transforms into ‘
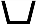
’ shape once the entire polymer has been etched away along its thickness.

Now, if the etching rate of the polymer, which depends on the etching agent and its strength, is *x* nm/s (~2 nm/s for PMMA A4 in the non-agitated acetone) and the first stage of etching is conducted for *t* seconds, then the polymer is etched by a length of *xt* nm in all directions. Thus, upon completion of the first stage of developing, the width of the etch profile at the top layer of the polymer (*W*_T_) becomes:

$W_{T}= A_{2}B_{2} =w_{0 }+ 2xt$ (S1)

For calculating the width at the bottom layer of the polymer (*W*_B_), we need to consider the same assumption of equal etching (*xt* nm) in all directions. This implies: *A*_1_*A*_2_*=O*_1_*O*_3_*= xt*. However, once the bottom of the etch trench touches Si at the point O_2_ at P_B_P_B_ plane, the polymer is etched in horizontal direction only. So, extrapolating the etch profile in Si, we get:

*O*_2_*C=O*_2_*D=O*_2_*O*_3_. (S2)

Thus, we can show that *O*_2_*O*_3_ *= W*_B_*/2*. Since the polymer thickness *OO_2_=H*, thus, we can write:

$H=h_{0}+O_{1}O_{2}=h_{0}+\left(O_{1}O_{3}-O_{2}O_{3}\right)=h_{0}+\left(xt-\frac{W_{B}}{2}\right)$ (S3)

or:

$W_{B}=2\left(h_{0}+xt-H\right)$ (S4)

Now, the slope of the sidewall of the polymer groove can be given as follows:

(S5)

Replacing *W*_B_ and *W*_T_ in equation (S5) in terms of *h*_0_, *xt* and *H* using Equations (S1) and (S4), we get the following expression for the slope:

(S6)

**Supplementary material 2. Throughput Calculation**

In electrolithography process, the minimum possible gap between two adjacent lines is twice the thickness of the polymer. To calculate the throughput of this process a 100 μm × 100 μm area is considered. If polymer thickness is 200 nm, then 200 lines, having width of 100 nm and length 100 μm, can be drawn in the assumed area. Considering a reasonable AFM tip speed of 50 μm/s while drawing the line, and 100 μm/s while moving from one line to the next, the time required to draw each line is 2 s. So the total time required to pattern an area of size 10^4^ μm^2^ is approximately 400 s. Therefore, the calculated throughput using AFM tip is approximately 10^5^ μm^2^/hour at the highest density of the written pattern.

If the probe tip diameter is bigger and the feature sizes are larger (> 1 μm) then the probes can be moved 10 times faster than the aforementioned speed. So, the throughput will also then become 10 times higher, i.e., 10^6^ μm^2^/h. On other hand, if very high currents are passed into very thick Cr films using large sized probes, the liquid material below the cathode probe can be removed from a large area in a very short time (e.g., rings having diameter of 200 µm form in 2 s.^28^ In such a case, the tip’s effective writing speed is ~ 3×10^8^ µm^2^/h. Thus, if the tip is moved very fast (say, at 1 mm/s) while it is not *writing* (i.e. while moving the tip from one line to the next line), the net throughput will be approximately 10^9^ µm^2^/h. This corresponds to the highest throughput achieved using a single tip. Therefore, this value of throughput can be improved drastically by using multiple tip technique.
